# Association between environmental exposure to perchlorate, nitrate, and thiocyanate and serum α-Klotho levels among adults from the National Health and nutrition examination survey (2007–2014)

**DOI:** 10.1186/s12877-022-03444-2

**Published:** 2022-09-12

**Authors:** Yu Yao, Gao-yan He, Xiao-juan Wu, Chao-ping Wang, Xiao-bin Luo, Yong Zhao, Ying Long

**Affiliations:** Department of Respiratory and Critical Care Medicine, Suining Central Hospital, No. 127, Desheng West Road, Chuanshan District, Suining, 629000 China

**Keywords:** Environmental exposure, Perchlorates, Nitrates, Thiocyanate, α-Klotho, Aging

## Abstract

**Background & aims:**

Aging is a pathophysiological process driven by a diverse set of complex biological processes, and environmental pollution plays an important role in this process. This study aimed to explore the association between serum α-Klotho levels and urinary perchlorate, nitrate, and thiocyanate levels.

**Methods:**

This secondary dataset analysis included 4875 participants (mean age, 57.69 year; male, 49.58%; non-Hispanic White, 47.67%) from the US National Health and Nutrition Examination Survey (2007–2014). Enzyme-linked immunosorbent assay was used to quantify α-Klotho levels, and ion chromatography coupled with electrospray tandem mass spectrometry was used to quantify thiocyanate, nitrate, and perchlorate levels. Multivariate linear regression models were applied to estimate the association between perchlorate, nitrate, and thiocyanate levels and serum α-Klotho levels.

**Results:**

Urinary thiocyanate levels were negatively associated with α-Klotho levels (*β* = − 0.006; 95% confidence interval, − 0.010 to − 0.003; *P* = 0.0004) after adjusting for age, sex, body mass index, race, alcohol consumption, estimated glomerular filtration rate, underlying disease, physical activity, smoking status, usual energy intake, and urinary creatinine and serum cotinine levels and mutual adjustment of urinary perchlorate, urinary nitrate, and urinary thiocyanate levels. The α-Klotho level in participants in the highest quartile was higher by 50.567 ng/mL (*β* = 50.567; 95% confidence interval, 14.407 to 86.726; *P* = 0.009) than that in participants in the lowest quartile of urinary perchlorate. A linear relationship was observed between urinary thiocyanate and α-Klotho levels.

**Conclusions:**

Urinary thiocyanate levels were negatively associated with serum α-Klotho levels. Urinary thiocyanate should be further investigated as a potential mediator of aging and age-related diseases.

**Supplementary Information:**

The online version contains supplementary material available at 10.1186/s12877-022-03444-2.

## Introduction

Aging is a pathophysiological process driven by a diverse set of complex biological processes associated with deteriorating physiological systems and accompanied by adverse health outcomes. The Klotho protein, encoded by the *Klotho* gene, which was originally identified as a gene that exerts antiaging effects, is expressed in various tissues, including the kidney, lung, choroid plexus, brain, parathyroid glands, and skeletal muscle [1–7]. There are three subfamilies of Klotho: α-, β-, and γ-Klotho. The word “Klotho” generally represents α-Klotho [8]. The decrease in Klotho levels contributes to the occurrence and development of many diseases, indicating that it acts on target tissues and plays a role in hormonal function [1, 2, 9–11].

Environmental pollutant exposure plays an important role in the acceleration of aging and the high morbidity and mortality rates of age-related diseases [12]. Thiocyanate, perchlorate, and nitrate are found ubiquitously in the environment, leading to broad human exposure and primary uptake through the food web, cigarette smoke, working in cyanide-related industries, and drinking water [13, 14]. These three chemicals have been reported as major thyroid-disrupting chemicals, have been known as sodium/iodide symporter inhibitors, and can cause thyroid dysfunction [13]. The hypothalamic–pituitary–thyroid axis is related with the aging process [15]. Moreover, several studies have reported that changes in thyroid-related hormones are associated with aging [16, 17]. However, the association between exposure to these three chemicals (thiocyanate, perchlorate, and nitrate) and serum α-Klotho levels remains unknown. Accordingly, our study aimed to explore the association between serum α-Klotho levels and urinary perchlorate, nitrate, and thiocyanate levels.

## Materials and methods

### Study design

This cross-sectional study collected data from four cycles of the US National Health and Nutrition Examination Survey (NHANES) between 2007 and 2014. NHANES was conducted by the National Center for Health Statistics (NCHS) at the US Center for Disease Control. Health survey interviews and physical examinations were conducted with a nationally representative sample of noninstitutionalized individuals in the United States. A multistage stratified sampling design with oversampling for certain subgroups was used to obtain health information in the survey using questionnaires about demographic characteristics and health history, and blood and urine samples were obtained during physical examinations. Data were collected for a 2-year survey cycle. Written consent was obtained from all participants, and this study was approved by the NCHS Ethics Review Board (Protocol #2005-06, Protocol #2011-17).

### Participants

Participants aged 40–79 years in the NHANES (2007–2014) with α-Klotho results were included in this study because the α-Klotho level was more susceptible and detectable in participants in this age group. The exclusion criterion was as follows: weak/failing kidney. The participants were divided into four groups according to quartiles [8].

### Variables

An enzyme-linked immunosorbent assay (ELISA) was adopted to quantify α-Klotho levels. The reference range was 285.8–1638.6 (mean, 698.0) pg/mL [17]. Briefly, wells of microtiter plates were coated with 67G3 monoclonal antibody in carbonate buffer and then blocked with bovine serum albumin in phosphate-buffered saline. The mixtures were loaded, incubated, and added with horseradish peroxidase. The reaction was stopped, and absorbance at 450 nm was measured with subtraction at 570 nm using an ELISA plate reader. The plates were rinsed with a washing buffer after each step. A standard curve was established with serial dilutions of recombinant α-Klotho.

Ion chromatography and electrospray tandem mass spectrometry were used to quantify thiocyanate, nitrate, and perchlorate levels in human urine specimens. Urinary creatinine level was measured using an enzymatic method in which creatinine was converted to creatine, which was then acted upon by creatinase to form sarcosine and urea. Sarcosine oxidase was used to convert sarcosine to glycine and hydrogen peroxide, and hydrogen peroxide reacted with the chromophore in the presence of peroxidase to produce a color product that was measured at 546 nm (secondary wavelength, 700 nm).

Underlying diseases, including congestive heart failure, coronary heart disease, heart attack, stroke, liver condition, and cancer or malignancy, were recorded as potential confounders, which may affect α-Klotho levels [10, 11, 13, 14, 18]. Other potential confounders included age, sex, race (Mexican American/other Hispanic/non-Hispanic White/non-Hispanic Black/other races), estimated glomerular filtration rate (eGFR), physical activity, usual energy intake, smoking status (minimum of 100 cigarettes in lifetime), alcohol consumption (minimum of 12 alcoholic drinks/year), body mass index (BMI), and physical activity. Obesity was defined as a BMI ≥ 30 kg/m^2^. Vigorous activity was defined as work with large increases in breathing or heart rate for at least 10 min in 1 week. Moderate activity was defined as work with small increases in breathing or heart rate at least 10 min in 1 week. Inactivity was defined as no vigorous activity and no moderate activity.

### Statistical analyses

Continuous variables are presented as mean and standard error. Categorical variables are presented as number and proportion. The chi-square or Kruskal–Wallis test was used for categorical and continuous variables without normal distribution, and analysis of variance was used for continuous variables. Summary characteristics provided nationally representative estimates and were adjusted for the complex sampling design, in which we constructed two multiple linear regression models with adjustments for possible baseline data imbalances. No covariates were adjusted in the crude model. Age, sex, BMI, race, alcohol consumption, eGFR, underlying disease, physical activity, smoking status, usual energy intake, and urinary creatinine level were adjusted, and mutual adjustment of urinary perchlorate, nitrate, and thiocyanate levels was performed in the adjusted model. Subgroup and interaction analyses were performed to examine the association between urinary thiocyanate level and α-Klotho level stratified by race (Mexican American vs. other Hispanics vs. non-Hispanic White vs. non-Hispanic Black vs. other races), sex (male vs. female), age (< 65 years vs. ≥65 years), obesity (obese vs. nonobese), alcohol consumption (≥12 alcoholic drinks/year vs. < 12 alcoholic drinks/year), physical activity (vigorous activity vs. moderate activity vs. inactivity), and smoking status (≥100 cigarettes in lifetime vs. < 100 cigarettes in lifetime). Smooth curve fitting was used to examine whether the independent variable was partitioned into intervals, and segmented regression and log-likelihood ratio tests were performed to determine whether a threshold existed. All statistical analyses were performed using R-project (http://www.R-project.org) and EmpowerStats (http://www.empowerstats.com). Full sample 2-year mobile examination center exam weight was divided by the number of cycles to recalibrate; then, all estimates were calculated using these recalibrated weights based on the analytical guidelines edited by the NCHS. *P*-value < 0.05 indicated statistical significance.

## Results

In total, 4875 participants (mean age, 57.69 years; male, 49.58%; non-Hispanic White, 47.67%) were enrolled (Table 1).

**Table 1:** Baseline characteristics of 4875 participants from the NHANES (2007–2014)

Characteristics	Total (*n* = 4875)	Thiocyanate	Thiocyanate	Thiocyanate	Thiocyanate	*P*
		Q1 (*n* = 1217)	Q2 (*n* = 1212)	Q3 (*n* = 1223)	Q4 (*n* = 1223)	
BMI (kg/m^2^)	4875	28.84 ± 5.88	29.79 ± 6.09	30.48 ± 6.75	28.92 ± 6.57	< 0.001
Sex						
Male	2417	468 (38.46%)	594 (49.01%)	642 (52.49%)	713 (58.30%)	< 0.001
Female	2458	749 (61.54%)	618 (50.99%)	581 (47.51%)	510 (41.70%)	
Age (years)^a^	4875	59.69 ± 11.09	58.72 ± 11.36	57.56 ± 10.63	54.82 ± 9.87	< 0.001
≥12 alcoholic drinks/year						< 0.001
Yes	3349	720 (59.16%)	794 (65.51%)	884 (72.28%)	951 (77.76%)	
No	1319	434 (35.66%)	366 (30.20%)	298 (24.37%)	221 (18.07%)	
Missing	207	63 (5.18%)	52 (4.29%)	41 (3.35%)	51 (4.17%)	
Race						< 0.001
Mexican American	772	259 (21.28%)	216 (17.82%)	187 (15.29%)	110 (8.99%)	
Other Hispanic	525	178 (14.63%)	152 (12.54%)	118 (9.65%)	77 (6.30%)	
Non-Hispanic White	2324	456 (37.47%)	565 (46.62%)	626 (51.19%)	677 (55.36%)	
Non-Hispanic Black	948	191 (15.69%)	211 (17.41%)	234 (19.13%)	312 (25.51%)	
Other races	306	133 (10.93%)	68 (5.61%)	58 (4.74%)	47 (3.84%)	
Perchlorate (ng/mL)	4875	4.02 ± 6.45	5.18 ± 8.93	6.41 ± 10.04	5.68 ± 17.53	< 0.001
Nitrate × 10^3^ (ng/mL)	4875	34.83 ± 38.35	49.10 ± 42.43	60.60 ± 48.00	67.49 ± 46.20	< 0.001
Urinary creatinine (mg/dL)	4875	82.38 ± 63.92	106.42 ± 66.53	124.95 ± 68.14	131.51 ± 73.43	< 0.001
eGFR (mL/min/1.73 m^2^)	4875	81.62 ± 19.03	82.33 ± 19.35	82.36 ± 17.58	82.69 ± 17.79	0.570
α-Klotho (pg/mL)	4875	874.97 ± 320.04	855.57 ± 295.41	860.18 ± 297.41	840.43 ± 290.88	0.103
Smoked at least 100 cigarettes in lifetime	4875					< 0.001
Yes	2449	423 (34.76%)	502 (41.42%)	555 (45.38%)	969 (79.23%)	
No	2426	794 (65.24%)	710 (58.58%)	668 (54.62%)	254 (20.77%)	
Usual energy intake, kcal/d	4875	118.79 ± 168.05	121.23 ± 165.29	132.60 ± 216.95	151.51 ± 209.20	< 0.001
Congestive heart failure	4875					
Yes	149	44 (3.62%)	27 (2.23%)	35 (2.86%)	42 (3.43%)	0.182
No	4727	1173 (96.38%)	1185 (97.77%)	1188 (97.14%)	1181 (96.57%)	
Coronary heart disease	4875					0.441
Yes	205	45 (3.70%)	60 (4.95%)	35 (2.86%)	52 (4.25%)	
No	4670	1172 (96.30%)	1152 (95.05%)	1188 (97.14%)	1171 (95.75%)	
Heart attack	4875					0.075
Yes	221	52 (4.27%)	53 (4.37%)	48 (3.92%)	71 (5.81%)	
No	4654	1165 (95.73%)	1159 (95.63%)	1175 (96.08%)	1152 (94.19%)	
Stroke	4875					0.419
Yes	198	57 (4.68%)	42 (3.47%)	45 (3.68%)	46 (3.76%)	
No	4677	1160 (95.32%)	1170 (96.53%)	1178 (96.32%)	1177 (96.24%)	
Liver condition	4875					0.262
Yes	213	50 (4.11%)	43 (3.55%)	58 (4.74%)	62 (5.07%)	
No	4662	1167 (95.89%)	1169 (96.45%)	1165 (95.26%)	1161 (94.93%)	
Cancer or malignancy	4875					0.411
Yes	581	140 (11.50%)	150 (12.38%)	158 (12.92%)	133 (10.87%)	
No	4294	1077 (88.50%)	1062 (87.62%)	1065 (87.08%)	1090 (89.13%)	
Physical activity	4875					< 0.001
Vigorous activity	858	157 (12.90%)	201 (16.58%)	201 (16.43%)	299 (24.45%)	
Moderate activity	1069	248 (20.38%)	270 (22.28%)	292 (23.88%)	259 (21.18%)	
Inactivity	2948	812 (66.72%)	741 (61.14%)	730 (59.69%)	665 (54.37%)	

*BMI* body mass index, *SE* standard error, *eGFR* estimated glomerular filtration rate

The results of the multiple linear regression analysis of the association between urinary perchlorate, nitrate, and thiocyanate levels and α-Klotho levels are shown in Table 2. There was a significant and negative unadjusted association between urinary thiocyanate and serum α-Klotho levels (*β* = − 0.005, *P =* 0.0002), and the association remained after adjusting for all covariates (*β* = − 0.006, *P =* 0.0004). In the adjusted model, the α-Klotho level decreased by 0.006 ng/mL with every one-unit increase in the urinary thiocyanate level. The α-Klotho level in participants in the highest quartile of urinary perchlorate was higher by 50.567 ng/mL than that in participants in the lowest quartile. However, no significant associations were found between urinary nitrate levels and serum α-Klotho levels and between urinary thiocyanate tertiles and serum α-Klotho levels.

**Table 2:** Association between urinary thiocyanate, nitrate, and perchlorate levels and α-Klotho levels assessed using multiple linear regression models (*n* = 4.875)

Urinary biomarkers	*n*	Crude model *β* (95% CI)	*P*	*P* for trend	Adjusted model^a^ *β* (95% CI)	*P*	*P* for trend
Thiocyanate (ng/ml)	4875			< 0.001			0.007
Q1	1217	Reference			Reference		
Q2	1212	−5.183 (−37.650, 27.284)	0.76		2.114 (−28.049, 32.276)	0.89	
Q3	1223	−21.587 (−59.246, 16.072)	0.27		−9.407 (−45.380, 26.567)	0.61	
Q4	1223	− 39.151 (−72.874, −5.429)	0.03		−33.240 (−69.913, 3.432)	0.08	
Thiocyanate levels		−0.005 (−0.008, − 0.003)	0.0002		−0.006 (− 0.010, − 0.003)	0.0004	
Nitrate × 10^−3^ (ng/mL)	4875			0.02			0.66
Q1	1256	Reference			Reference		
Q2	1260	−7.260 (−40.930–26.409)	0.67		−1.080 (−36.328–34.167)	0.95	
Q3	1257	11.929 (−23.801–47.659)	0.52		24.678 (−16.340–65.696)	0.25	
Q4	1266	−32.880 (−70.037–4.277)	0.09		−15.560 (−61.376–30.255)	0.51	
Nitrate × 10^−3^ levels	4875	−0.000 (−0.000 − 0.000)	0.19		–0.000 (−0.00–0.000)	0.50	
Perchlorate (ng/mL)	4875			0.63			0.11
Q1	1249	Reference			Reference		
Q2	1268	8.954 (−25.792–43.700)	0.62		26.810 (−6.710–60.331)	0.13	
Q3	1261	−19.580 (−49.561–10.401)	0.21		14.088 (−15.435–43.611)	0.36	
Q4	1261	15.435 (−22.211–53.081)	0.42		50.567 (14.407–86.726)	0.009	
Perchlorate levels		0.038 (−0.585–0.661)	0.91		0.389 (−0.047–0.825)	0.09	

*CI* confidence interval

^a^Adjusted for age, sex, body mass index, race, alcohol consumption, estimated glomerular filtration rate, underlying disease, physical activity, smoking status, usual energy intake, and urinary creatinine and serum cotinine levels and mutually adjusted for urinary perchlorate, urinary nitrate, and urinary thiocyanate levels

Subgroup and interaction analyses for the association between exposure to thiocyanate and serum α-Klotho levels stratified by various risk factors are shown in Table 3. The negative association between urinary thiocyanate levels and serum α-Klotho levels was significant in most subgroups. However, the association was not significant in Mexican Americans, other races, non-Hispanic Blacks, male participants, participants aged ≥65 years, participants with vigorous activity, and nonobese participants. We found that sex and thiocyanate level had interactive effects on serum α-Klotho levels.

**Table 3:** Association between urinary thiocyanate levels and α-Klotho levels stratified by subgroup and interaction analyses (*n* = 4.875)

Subgroups	Total (*n* = 4.875)	*β* (95% CI)	*P*	Interaction *P*
Mexican American	772	0.00 (− 0.01 − 0.01)	0.86	0.68
Other Hispanic	525	−0.01 (− 0.02–0.01)	0.49	
Non-Hispanic White	2324	–0.01 (−0.01 to − 0.00)	0.002	
Non-Hispanic Black	948	−0.00 (− 0.01–0.00)	0.22	
Other races	306	−0.01 (− 0.03 to − 0.00)	0.02	
Male	2417	− 0.00 (− 0.01–0.00)	0.18	0.003
Female	2458	−0.01 (− 0.02 to − 0.01)	< 0.0001	
< 65 years	3446	− 0.01 (− 0.01 to − 0.00)	< 0.0001	0.40
≥65 years	1429	− 0.00 (− 0.01 − 0.01)	0.72	
Obesity	2915	–0.01 (− 0.01 to − 0.00)	0.0001	0.16
Non-obesity	1960	− 0.00 (− 0.01–0.00)	0.09	
≥12 alcoholic drinks/year	3349	− 0.01 (− 0.01 to − 0.00)	0.0004	0.73
< 12 alcoholic drinks/year	1319	− 0.01 (− 0.01 to − 0.00)	0.03	
Vigorous activity	858	− 0.00 (− 0.01–0.00)	0.38	0.27
Moderate activity	1069	− 0.01 (− 0.02 to − 0.00)	0.003	
Inactive	2948	− 0.01 (− 0.01 to − 0.00)	0.0009	
≥100 cigarettes in lifetime	2449	− 0.01 (− 0.01 to − 0.00)	0.0021	0.05
< 100 cigarettes in lifetime	2462	− 0.01 (− 0.02 to − 0.00)	0.0023	

*CI* confidence interval

**Fig. 1:**
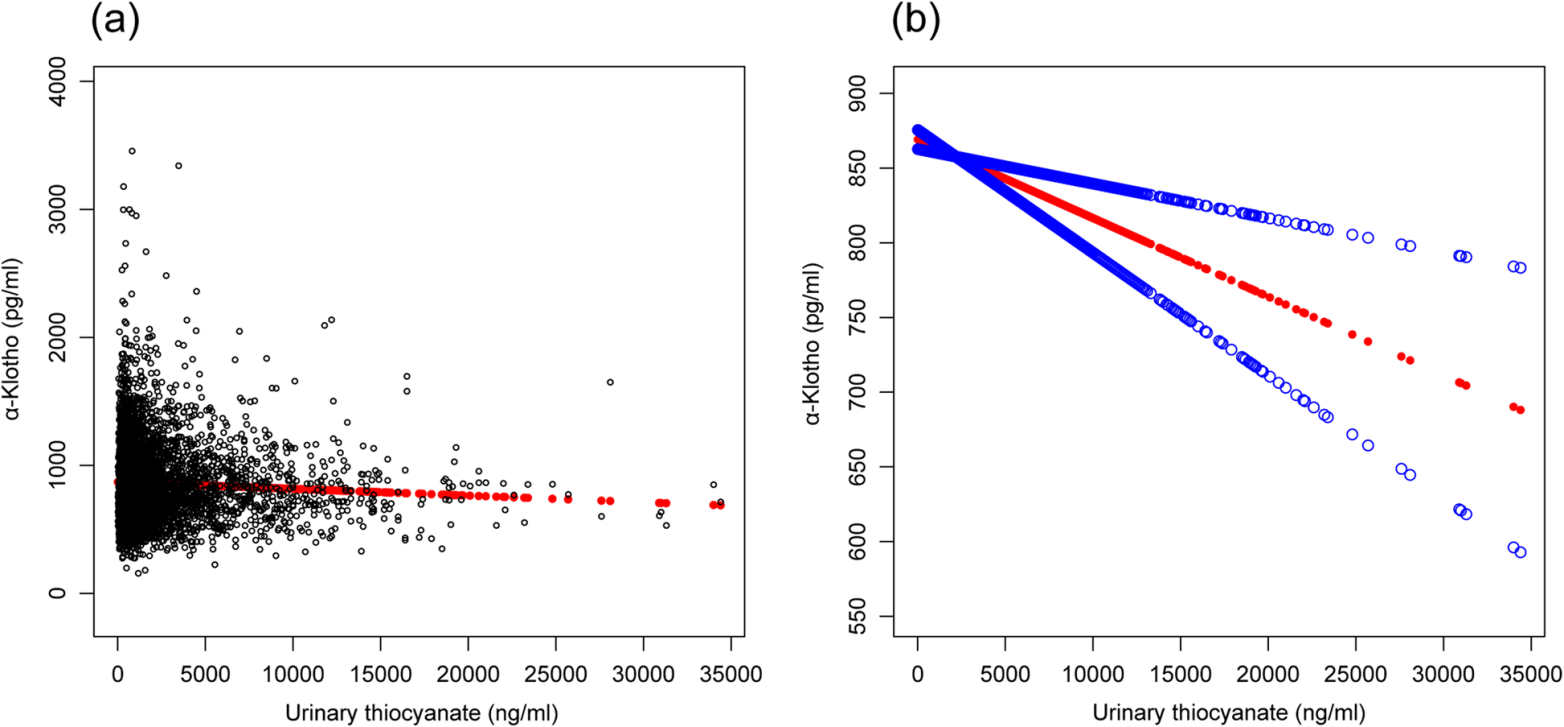
**a** Scatter curve of the association between urinary thiocyanate levels and serum α-Klotho levels after adjusting for age; sex; body mass index; race; alcohol consumption; and urinary creatinine, perchlorate, and nitrate levels. **b** Smooth curve of the association between urinary thiocyanate levels and serum α-Klotho levels after adjusting for the aforementioned variables

We found a linear relationship between urinary thiocyanate and serum α-Klotho levels after adjusting for all covariates (Fig. 1).

## Discussion

To the best of our knowledge, this is the first study to investigate the association between urinary perchlorate, nitrate, and thiocyanate levels and serum α-Klotho levels. We found that urinary thiocyanate levels were inversely associated with serum α-Klotho levels. Compared with that in the lowest quartile of urinary perchlorate, the α-Klotho level in the highest quartile was higher by 50.567 ng/mL, regardless of all covariates. No significant associations were observed between serum α-Klotho and urinary nitrate levels and urinary perchlorate levels. Moreover, a linear relationship was observed between urinary thiocyanate and α-Klotho levels.

We found sex-related differences in the association between urinary thiocyanate and serum α-Klotho levels. The association was only significant in female participants. Furthermore, there was a significant interactive effect of sex and thiocyanate levels on serum α-Klotho levels. Several studies have reported sex-related differences in aging in humans [18]. Moreover, women have higher α-Klotho levels than men [19–21]. These findings illustrate that sex-related differences play an important role in the association between thiocyanate level and aging. In this study, we observed no significant association between urinary thiocyanate and α-Klotho levels in Mexican American, other Hispanic, and non-Hispanic Black populations. Thiocyanate was also a biomarker of cyanide exposure from tobacco smoke [22]. Previous studies have shown racial differences, which are related to different tobacco brands, differences in pharmacokinetics related to cytochrome P450 activity (CYP2A6), and smoking methods/habits [16]. Based on our findings, there was no significant association between urinary thiocyanate and α-Klotho levels in the elderly. The elderly may have lower α-Klotho levels, owing to which urinary thiocyanate plays a minor role in α-Klotho levels. This study also found that the association between urinary thiocyanate and α-Klotho levels was not significant in nonobese participants. A previous study reported that a higher exposure to urinary thiocyanate was associated with a higher risk of obesity, suggesting that nonobese participants have lower urinary thiocyanate levels [23]. Furthermore, we found that the association between urinary thiocyanate levels and serum α-Klotho levels was not significant in participants with vigorous activity. A relatively recent study showed that serum α-Klotho levels increased with the increase in vigorous activity time, suggesting that individuals with vigorous activity have higher serum α-Klotho levels than individuals without vigorous activity. Vigorous activity may reduce the risk of serum α-Klotho levels induced by urinary thiocyanate. After adjustment of eGFR, we found no significant association between urinary perchlorate levels and serum α-Klotho levels. Klotho is a transmembrane protein and expressed particularly high in the kidney, which regulates calcium metabolism and parathyroid hormone synthesis [24]. Furthermore, several studies have suggested that patients with kidney diseases have low level of α-Klotho [25, 26]. Therefore, urinary perchlorate levels was not an independent factor which has an impact on serum α-Klotho levels.

Here, we found that urinary thiocyanate levels were inversely associated with serum α-Klotho levels. The underlying mechanisms behind this inverse association require further investigation. Thiocyanate can competitively inhibit radioactive iodide uptake by the human sodium iodide symporter to cause thyroid dysfunction and thyroid-related hormone changes [13, 27–29], which play an important role in aging and many age-related diseases [30–32]. Exposure to thiocyanate from diet may cause an increased risk of protein carbamylation, which is a hallmark of aging in mammalian species [33, 34]. Moreover, thiocyanate is also a biomarker for cyanide exposure in tobacco smoke and is primarily formed in the body as a metabolite of cyanide from tobacco smoke. Cigarette smoke is an important accelerator of the aging process, both directly through complex mechanisms mediated by excessive formation of free radicals and indirectly by favoring various pathologies [35]. Thiocyanate can directly involved in the synthesis of hypothiocyanous acid, which induces cellular damage by targeting thiols [36]. Therefore, the negative association between thiocyanate and α-Klotho levels may reflect the effects of cigarette smoke, protein carbamylation, and thyroid dysfunction on aging.

Our study has some limitations. First, data were obtained from single measurements of perchlorate, nitrate, and thiocyanate in urine samples, which might not reflect the effects of long-term exposure among participants. Second, as a cross-sectional study, its temporality and residual confounding were unavoidable. Third, residual confounders likely remained, although many potential confounders were adjusted in this study. The strengths of this study include the use of a large representative and multiracial sample of the US population; involvement of stratification analyses of perchlorate, nitrate, and thiocyanate levels; and use of segmented regression and log-likelihood ratio tests and smooth curve fitting to enhance the robustness of the results and identify special populations.

## Conclusion

Our study showed that urinary thiocyanate levels were inversely associated with serum α-Klotho levels, regardless of the covariates. No significant association was observed between serum α-Klotho levels and urinary nitrate and perchlorate levels. These findings highlight the need to longitudinally evaluate the effects of environmental exposure to perchlorate, nitrate, and thiocyanate on aging in humans. Further investigations are required to focus on the potential mechanisms underlying the association between exposure to these chemicals and aging.

## Supplementary Information

**Additional file 1.**

## Data Availability

The datasets generated and/or analyzed during the current study are available in the NHANES database repository: https://wwwn.cdc.gov/nchs/nhanes/ContinuousNhanes/Default.aspx?BeginYear=2013 and https://wwwn.cdc.gov/nchs/nhanes/ContinuousNhanes/Default.aspx?BeginYear=2011, https://wwwn.cdc.gov/nchs/nhanes/continuousnhanes/default.aspx?BeginYear=2009, https://wwwn.cdc.gov/nchs/nhanes/continuousnhanes/default.aspx?BeginYear=2007.
