# Supplementary figures and images for: Association between environmental exposure to perchlorate, nitrate, and thiocyanate and serum α-Klotho levels among adults from the National Health and nutrition examination survey (2007–2014)
Source: BMC Geriatr. 2022 Sep 12;22:740. doi: 10.1186/s12877-022-03444-2 (PMC9465863; doi:10.1186/s12877-022-03444-2)

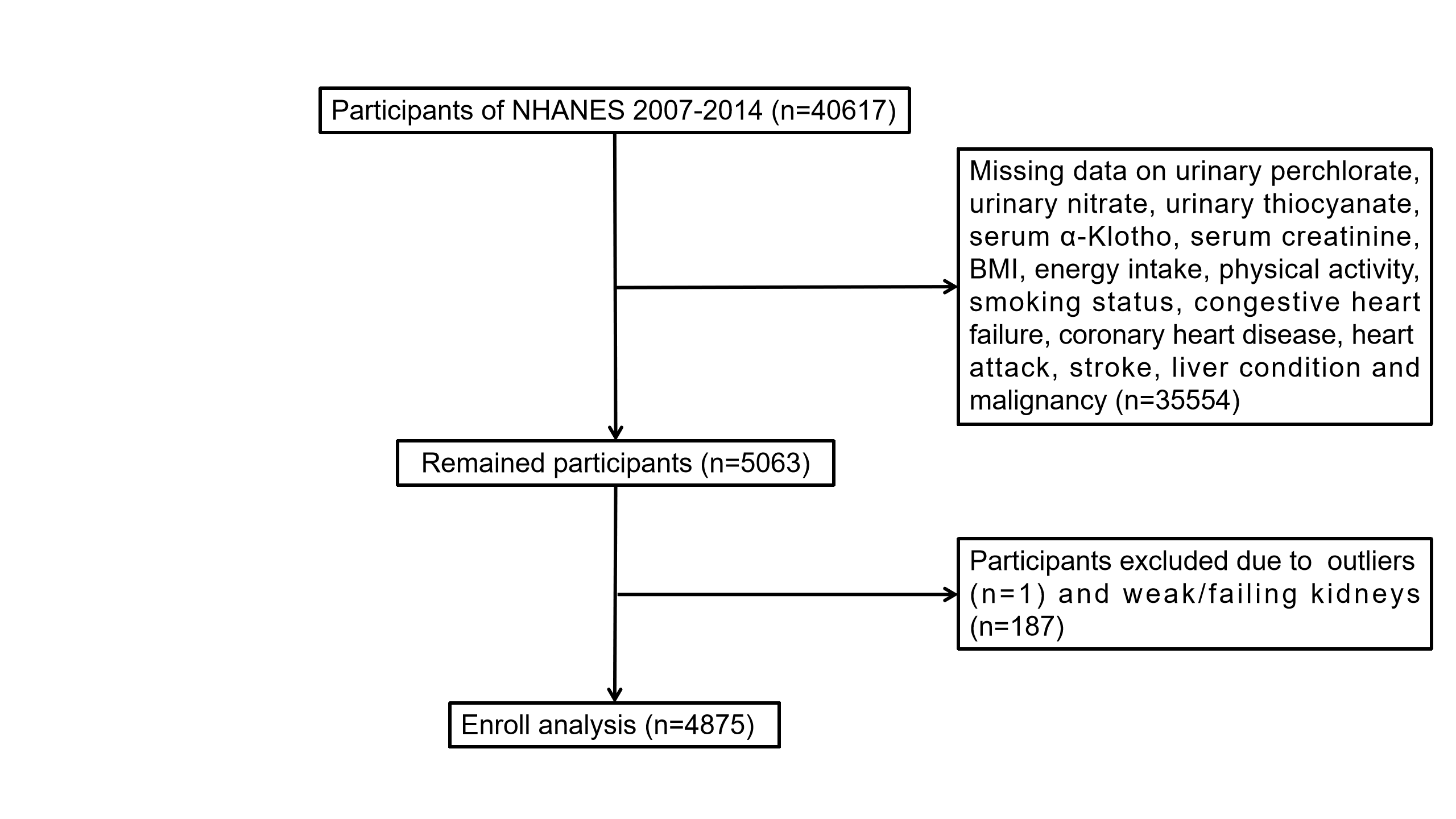

Supplement: Supplementary file 1 — Additional file 1. [file 12877_2022_3444_MOESM1_ESM.tif]
